# Efeitos Cardiovasculares do Diterpeno Manool em Ratos Normotensos e Hipertensos

**DOI:** 10.36660/abc.20200552

**Published:** 2020-10-13

**Affiliations:** 1 Universidade Federal de Goiás GoiâniaGO Brasil Universidade Federal de Goiás, Goiânia, GO - Brasil

**Keywords:** Doenças Cardiovasculares, Hipertensão, Ratos, Plantas, Diterpeno Manool, Plantas Medicinais

A Organização Mundial da Saúde estima que as doenças cardiovasculares (DCVs) sejam a principal causa de morte no mundo, sendo responsáveis por 17,5 milhões de mortes por ano, número que deve ultrapassar 23,6 milhões até 2030.^1^ Da mesma forma, no Brasil, as doenças do sistema circulatório causaram 30,68% do total de mortes em 2014.^2^

A hipertensão é quantitativamente o fator de risco mais importante para DCVs, como acidente vascular cerebral, infarto do miocárdio e insuficiência cardíaca.^3^ A prevalência global de hipertensão em adultos com 18 anos ou mais foi aproximadamente 22% em 2014. A alta incidência de hipertensão e outras DCVs também geram um importante problema econômico, pois são responsáveis por altas taxas de mortalidade e incapacidade na população economicamente ativa.^3^

É importante ressaltar que mesmo com a grande diversidade de medicamentos disponíveis atualmente, ainda existe um aumento na prevalência das DCVs. Isso enfatiza a importância de estudos com o objetivo de descobrir novas substâncias com efeitos anti-hipertensivos ou cardioprotetores, de baixo custo e poucos efeitos adversos. Nesse sentido, o potencial terapêutico de produtos derivados de plantas para DCVs foi documentado em estudos anteriores.^4^^–^^6^ De fato, alguns medicamentos já disponíveis comercialmente foram desenvolvidos a partir de substâncias encontradas anteriormente em plantas, como a digoxina, derivada da *D. lanata*, usada no tratamento da insuficiência cardíaca congestiva por muitas décadas, e a reserpina, derivada da *R. serpentina*, que foi um dos primeiros medicamentos utilizados no tratamento da hipertensão.^5^

Monteiro et al.,^7^ avaliaram o efeito do manool na pressão arterial no modelo animal de hipertensão renovascular dois rins um clipe (2K1C). O manool promoveu redução da pressão arterial sistólica em ratos normotensos e hipertensos. O efeito encontrado em animais hipertensos foi, pelo menos em parte, mediado pela via do óxido nítrico, uma vez que o pré-tratamento com o inibidor não-específico da óxido nítrico sintase, L-NAME, atenuou o efeito anti-hipertensivo do manool. Além disso, o manool promoveu um aumento nos níveis plasmáticos de nitrito / nitrato (NOx) em ratos hipertensos, mas não nos normotensos. Esses dados sugerem uma possível diferença nos mecanismos de ação do manool entre animais normotensos e hipertensos. Infelizmente, os autores não mostraram se as mudanças na pressão arterial foram acompanhadas por alterações da frequência cardíaca. De forma interessante, os autores demonstraram que o manool parece atuar no endotélio vascular. Foi observado que o manool causou vaso-relaxamento somente nos anéis aórticos com endotélio intacto de ratos normotensos. Entretanto, não foram demonstrados os efeitos do manool na aorta de ratos hipertensos.

O manool pertence à classe dos compostos diterpênicos e é encontrado em maiores concentrações na espécie *Salvia officinalis.*^8^ Há poucos estudos que avaliaram o efeito do manool no sistema cardiovascular. Entretanto, muitos estudos experimentais e clínicos têm demonstrado alguns efeitos benéficos de várias classes de diterpenóides para DCVs.^6^ Os diterpenóides podem induzir relaxamento vascular e diminuir a pressão arterial sistólica em ratos espontaneamente hipertensos^9^ e diurese e natriurese em ratos normotensos.^10^ Estudos clínicos demonstraram que esteviosídeo administrado por via oral reduz a pressão arterial sistólica e diastólica.^11^ Além disso, a administração intravenosa de diterpeno forscolina do tipo labdano reduz a pressão arterial diastólica e melhora a função ventricular esquerda em pacientes com miocardiopatia.^12^

Esses estudos apontaram os diterpenóides como um alvo potencial para o desenvolvimento de novos agentes terapêuticos cardiovasculares. Corroborando com estes estudos, Monteiro et al.,^7^ demonstraram que o manool, um diterpenóide labdano, também apresenta algumas ações benéficas no sistema cardiovascular. Além disso, este estudo fornece informações sobre os mecanismos de ação gerados pelo manool que podem servir de base para estudos futuros visando o desenvolvimento de novos fármacos para o tratamento das DCVs. Entretanto, novos estudos experimentais, incluindo outros modelos animais e estudos clínicos são essenciais para confirmar esta hipótese.
