# Supplementary material for: Efeitos Cardiovasculares do Diterpeno Manool em Ratos Normotensos e Hipertensos
Source: Arq Bras Cardiol. 2020 Oct 13;115(4):678–9. [Article in Portuguese] doi: 10.36660/abc.20200552 (PMC8386984; doi:10.36660/abc.20200552)
Supplement: Translation

# Translation: Cardiovascular Effects of the Diterpene Manool in Normotensive and Hypertensive Rats

**Affiliations:** 1 GoiâniaGO Brazil Universidade Federal de Goiás, Goiânia, GO - Brazil

**Keywords:** Cardiovascular Diseases, Hypertension, Rats, Plants, Diterpenes Manool, Plants, Medicinal

The World Health Organization estimates that cardiovascular diseases (CVD) are the leading cause of death globally, accounting for 17.5 million deaths per year, a number that is expected to grow to more than 23.6 million by 2030.^1^ Similarly, in Brazil, diseases of the circulatory system caused 30.68 % of the total deaths in 2014.^2^

Hypertension is quantitatively the most important risk factor for CVD, such as stroke, myocardial infarction, and cardiac failure.^3^ The global prevalence of hypertension in adults aged 18 years and over was around 22% in 2014. The high incidence of hypertension and other CVD also generates an important economic problem since they are responsible for high rates of mortality and disability in the economically active population.^3^

It is important to note that even with the great diversity of drugs currently available, there is still an increase in the prevalence of CVD. This emphasizes the importance of studies with purposes of discovering new substances with antihypertensive or cardioprotective effects, low cost, and few adverse effects. In this sense, the therapeutic potential of plant-derived products for treatment of CVD has been documented in previous studies.^4^^–^^6^ Indeed, some commercial drugs have been developed from substances found previously in plants, such as digoxin, derived from *D lanata*, used in the treatment of congestive heart failure for many decades, and the reserpine, derived from *R serpentine*, which was one of the first drugs used on the treatment of hypertension.^5^

Monteiro et al.^7^ evaluated the effect of manool on blood pressure in two-kidney one-clip (2K1C) renovascular hypertension animal model. The manool promoted a reduction in the systolic blood pressure in both normotensive and hypertensive rats. The effect found in hypertensive animals was, at least in part, mediated by nitric oxide pathways, since the pre-treatment with a nonspecific nitric oxide synthase inhibitor, L-NAME, attenuated the antihypertensive effect of manool. Additionally, manool promoted an increase in plasma nitrite/nitrate (NOx) levels in hypertensive, but not normotensive rats. This data suggest a possible difference in action mechanisms of the manool between normotensive and hypertensive animals. Unfortunately, the authors did not show whether the changes in blood pressure were accompanied by alterations of the heart rate. Interestingly, the authors demonstrated that manool seems to act on vascular endothelium. It was observed that manool evoked vasorelaxation only in endothelium-intact aortic rings from normotensive rats. However, it was not demonstrated the effects of manool in aorta of the hypertensive rats.

Manool belongs to the class of diterpene compounds and is found in higher concentrations in the species *Salvia officinalis*.^8^ There are very few studies directed to evaluate the effect of manool on the cardiovascular system. However, many experimental and clinical studies have shown the beneficial effects of several classes of diterpenoids for CVD.^6^ Diterpenoids promote vascular relaxation and reduction in systolic blood pressure in spontaneously hypertensive rats^9^ and diuresis and natriuresis in normotensive rats.^10^ Clinical studies demonstrated that stevioside administered orally reduces systolic and diastolic blood pressures.^11^ Also, intravenous administration of labdane-type diterpene forskolin reduces diastolic blood pressure and improved left ventricular function in patients with cardiomyopathy.^12^

These studies pointed out the diterpenoids as a potential target for the development of novel cardiovascular therapeutic agents. In this sense, Monteiro et al.^7^ showed that manool, a diterpenoid labdane also has some beneficial actions on the cardiovascular system. Besides, this study provides information on the mechanisms of action elicited by manool that can form a basis for future studies aiming at the development of new drugs for the treatment of CVD. However, further experimental studies, including other animal models and clinical studies are essential to confirm this hypothesis.
